# Polymer Nanocomposites via Click Chemistry Reactions

**DOI:** 10.3390/polym9100499

**Published:** 2017-10-11

**Authors:** Mehmet Arslan, Mehmet Atilla Tasdelen

**Affiliations:** Department of Polymer Engineering, Faculty of Engineering, Yalova University, 77200 Yalova, Turkey

**Keywords:** click chemistry, polymer nanocomposites, functional nanocomposites

## Abstract

The emerging areas of polymer nanocomposites, as some are already in use in industrial applications and daily commodities, have the potential of offering new technologies with all manner of prominent capabilities. The incorporation of nanomaterials into polymeric matrix provides significant improvements, such as higher mechanical, thermal or electrical properties. In these materials, interface/interphase of components play a crucial role bringing additional features on the resulting nanocomposites. Among the various preparation strategies of such materials, an appealing strategy relies on the use of click chemistry concept as a multi-purpose toolbox for both fabrication and modulation of the material characteristics. This review aims to deliver new insights to the researchers of the field by noticing effective click chemistry-based methodologies on the preparation of polymer nanocomposites and their key applications such as optic, biomedical, coatings and sensor.

## 1. Introduction

Dispersing nano-sized reinforcing agents in the polymeric matrix yields polymer nanocomposites, and such materials exhibit superior physical, mechanical and thermal properties compared to their parent components. The leading properties of the constituents are inherently constructing the new materials as well as gaining additional properties. For the reinforcer and matrix materials, substantially different properties arise in, for instance, dimensional stability, Young’s modulus, gas barrier performance and fire retardancy that cannot be achieved by sole polymers or fillers [1,2]. Polymer nanocomposite technology has provided extensive insight on the nano-sized materials as unique handles of material design, recruiting nanoparticles, nanominerals, nanotubes, nanofibers, nanowires and fullerenes as reinforcing components [3,4]. A vast number of polymer matrices have been utilized as templates for nanocomposite fabrication [4]. Despite the fact that polymer nanocomposites exhibit great promise for development of potentially useful materials, the fabrication and processing of these materials is usually not straightforward. Important parameters, such as careful selection of polymer matrix, choosing appropriate nanofiller by considering entire nanocomposite system, the interactions between the components and their morphology as well as interfacial characteristics must be undertaken to achieve desirable end-goals of materials. The interfacing between the polymer matrix and the nanofiller and the interaction of individual components with themselves often necessitate careful analysis of the system and may require organic treatment and chemical modification of components and post-fabrication modification to impart desired properties [5]. In this sense, recently developed click chemistry reactions always have great relevance to enhance the properties of polymer nanocomposites. Besides their quantitative efficiency, the tolerance to a wide variety of functional groups and reaction conditions make them highly attractive for the nanocomposite preparation.

**Scheme 1:**
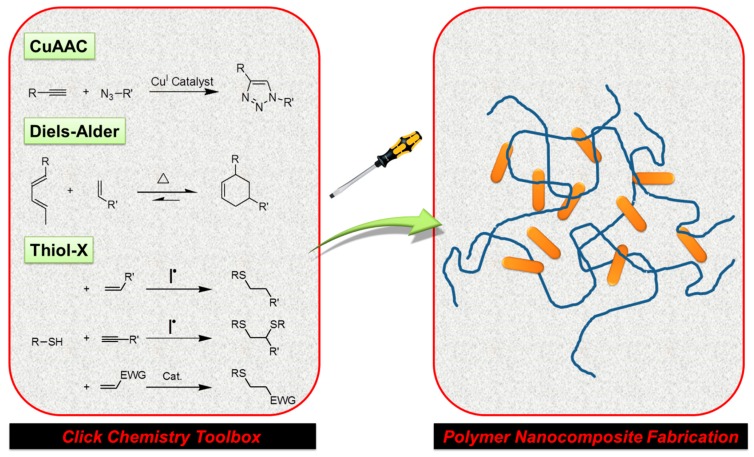
Representation of widely applied click reactions in polymer nanocomposite fabrication.

Click chemistry is a concept of highly efficient chemical reactions in which these reactions share some important common features: high reaction yields, benign reaction conditions and easily removable byproducts, stereospecific reaction coordinates and tolerance to diverse functional groups [6]. Due to allowing effective coupling of complementary functional groups, click reactions are not only utilized in organic [7] or medicinal [8] chemistry, but in fact, polymer science and material engineering [9] have facilitated these reactions and transformed them into convenient, versatile and reliable coupling procedures [10,11,12]. Though there are a handful of reactions that obey ‘click’ basis, elaborate choosing of building blocks may allow ample opportunities in design, fabrication and post-polymerization modification of polymer nanocomposites. In this report, we aim to highlight the click concept as a versatile tool in polymer nanocomposite fabrication and emphasize their potentials and limitations (Scheme 1). Commonly employed click reactions of copper(I)-catalyzed alkyne-azide cycloaddition (CuAAC) and metal-free click, Diels–Alder (DA) reaction, radical and nucleophilic thiol-ene reactions and thiol-yne reaction were primarily considered as click tools of nanocomposite preparation. Since there are excellent reviews on nucleophilic ring opening of epoxides and aziridines in polymer nanocomposite fabrication [13,14,15], these studies are not included in the current manuscript. The following studies are classified based on the type of click reaction that has been utilized in fabrication or modification and special emphasis is devoted to synthetic methodologies of manufacturing processes. 

## 2. Click Chemistry-Based Methodologies in Polymer Nanocomposite Fabrication

### 2.1. Polymer Nanocomposites via CuAAC and Metal-Free Click Reactions

The CuAAC click reaction facilitates the coupling of terminal alkynes and azides by affording 1,4-disubstituted triazoles in which the reaction is expedited by copper(I) catalyst. Because of the fairly unreactive nature of alkynes towards azides, reactions inevitably require metal catalyst in proper oxidation state (such as copper Cu(I) in the +1 oxidation state or Cu(II) with a combination of reducing agent) [16,17]. As a most prominent example of click chemistry, CuAAC reaction facilitates high selectivity, rapid and quantitative transformations, tolerance to diverse organic solvents and water, stability and orthogonality. Catalyst-free click reaction of azides with strain-promoted alkynes possesses significantly lowered activation energy and does not require any metal catalyst. Even though the strain-promoted alkynes are quite uncommon and expensive materials, metal-free click reaction (also referred to as strain-promoted azide-alkyne click chemistry (SPAAC) reaction) is now becoming an important click tool, especially if toxic metal catalyst is an issue [18]. 

**Figure 1:**
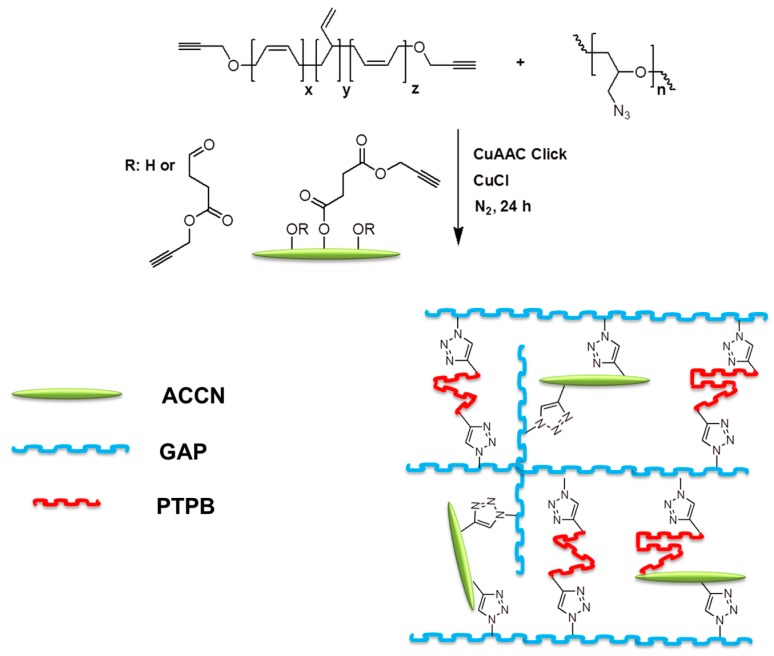
Formation of nanocrystal cellulose-based nanocomposites via CuAAC click reaction. Adapted with permission from [19]. Copyright © 2015 by The Royal Society of Chemistry.

In this regard, the CuAAC click reaction was utilized in fabrication of polymer nanocomposites of cellulose nanocrystals (CNCs) with polybutadiene and polyglycidyl polymers [19]. The cellulose nanocrystals have great interest as nanofillers; however, abundant hydrophilic hydroxyl groups often limit the dispersibility of CNCs in low polarity solvents. Most of the time, hydrophobic modification of CNCs allows improved compatibility of nanocrystals with polymer matrix. In the approach, propargyl-terminated polybutadiene (PTPB) and alkyne-modified cellulose nanocrystals (ACNCs) were effectively coupled with azide-functional glycidyl polymer (GAP) by using Cu(I)Cl catalyst (Figure 1). According to the mechanical analysis, the CNCs-reinforced nanocomposites showed increased mechanical properties than the sole GAP/PTPB material even by using one percent amount of reinforcer (1.0 wt % ACNC).

**Figure 2:**
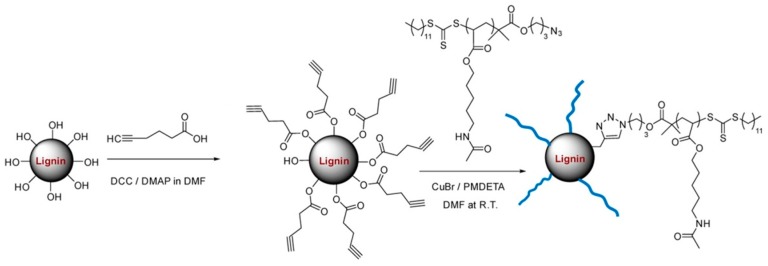
Modification and CuAAC click coupling of lignin in nanocomposite fabrication. Adapted with permission from [20]. Copyright © 2016 by the American Chemical Society.

Chung and co-workers recently reported an intriguing example of polymer nanocomposite fabrication by employing lignin as reinforcing component [20]. Lignin is a biopolymer which is second most abundant among the plant-based polymers and primarily constitutes the structural component of cell walls of vascular plants and some algae. The fabrication strategy included the synthesis of lignin-grafted polymer of 5-acetylaminopentyl acrylate (lignin-graft-PAA), which was prepared by CuAAC click coupling of alkyne-modified lignin and azide functional polymer (Figure 2). According to static tensile strength analysis, a nanocomposite of 15–20 wt % lignin content demonstrated the most optimal rubber-like and flexible properties. In addition, the material exhibited self-healing properties due to the high degree of hydrogen bonding of acetylamino groups. 

The polymer nanocomposites of inorganic nanoparticles (NPs) convey unique advantages. The multifunctional properties of inorganic nanoparticles such as magnetic, electronic, optical and catalyst features can be inherited by the composite material combining with enhanced mechanical properties [21,22]. Nevertheless, the intrinsic difficulty of dispersing inorganic NPs in organic polymer matrix is a key obstacle and requires appropriate modification of nanoparticles. Especially in optical applications of polymer nanocomposites, perfectly dispersed inorganic nanoparticles in organic polymer network is often required to maintain high visible transparency. In an attempt of fabricating highly transparent indium tin oxide (ITO)/epoxy nanocomposites, Siegel and co-workers utilized a combination of phosphate ligand coupling and CuAAC click reactions [23]. Polymer-modified nanoparticles were prepared by grafting the nanoparticle surface with alkyne-functionalized phosphate ligand, and subsequent Cu(I)Br-assisted click coupling of alkyne-terminated poly(glycidyl methacrylate). Optical nanocomposites were prepared by mixing polymer-modified NPs with diglycidyl ether bisphenol A (DGEBA), epoxy resin and trimethyl-1,6-hexanediamine curing agent. It was shown by ultraviolet-visible-near-infrared (UV-Vis-NIR) analysis that the prepared ITO-based nanocomposites exhibited >90% of visible light transparency and absorbed the UV light in the range of 300–400 nm. The nanocomposites also demonstrated low near-infrared light transmittance with increased IR shielding efficiency, upon increasing ITO concentration. In a similar organic modification of NPs, Vaia and co-workers prepared azide functional titanium dioxide (TiO_2_) NPs and further functionalized them with alkyne-terminated polystyrene via CuAAC click reaction [24]. A nanocomposite was fabricated by direct assembly of hybrid core-shell nanoparticles exhibiting a dielectric constant of 6.4 and a dielectric loss of 0.04 at 1 kHz.

The aluminum NPs with polyurethane (PU)-based polymer matrix were used in the preparation of nanocomposites for energetic application [25]. These nanocomposites are referred to as “nano-thermites” or “superthermites” that possess higher rate of energy release than the conventional thermites due to high surface area of the reactants in composite matrix. The fabrication method included the reaction of poly(glycidyl azide-*co*-tetramethylene glycol) with isophorone diisocyanate, in combination with a catalyst-free azide-alkyne cycloaddition of alkyne functional perfluoroacid. The differential scanning calorimetry (DSC) analysis indeed confirmed the thermite reaction and the resulting nanocomposites were presented as energetic materials with tailorable exothermic properties. 

Polymer nanocomposites of magnetic nanoparticles are of interest, since these materials can endow promising performance, such as in the removal of magnetic pollutant species from wastewater. In a study, Lakouraj et al. demonstrated the preparation of chitosan and thiacalix [4] arene-based nanocomposites for selective ion removal [26]. Firstly, chitosan was modified with magnetic Fe_3_O_4_ NPs, then azide groups were introduced onto the chitosan-Fe_3_O_4_ nanoparticle scaffold. The magnetic nanocomposite was obtained by CuAAC click coupling of alkyne functional thiacalix [4] arene. The fabricated nanocomposites provided efficient removal of toxic metal cations from wastewater. In another application of iron NPs, the Fe_3_O_4_ NPs/polycarbazole-based nanocomposites were synthesized from alkyne functional carbazole-based conjugated polymer and azide-terminated magnetic Fe_3_O_4_ NPs [27]. The obtained material was efficiently quenched by I^−^ ions, providing a reversible Hg^2+^ optical probe. The strategy of anchoring azide functional ligand onto nanoparticle surface was also exemplified in development of fluorescent polyurethane-ZnO hybrid nanocomposite coatings [28]. In a recent study, antibacterial thermoset nanocomposites containing silver NPs was successfully prepared by combination of simultaneous photoinduced electron transfer and CuAAC processes [29]. The photogenerated radicals not only reduce Cu(II) into Cu(I) activator to catalyst the CuAAC click reaction between multifunctional azide and alkyne molecules, but also they were simultaneously oxidized by silver(II) nitrate to generate silver NPs. The antibacterial activities of resulting nanocomposites were confirmed against gram-positive and -negative bacteria. Furthermore, the presence of silver nanoparticles in the thermoset matrix were also proven by transmission electron microscopy (TEM) with energy dispersive X-ray system analyzer (FEI TecnaiTM G2 F30, Eindhoven, The Netherlands). 

**Figure 3:**
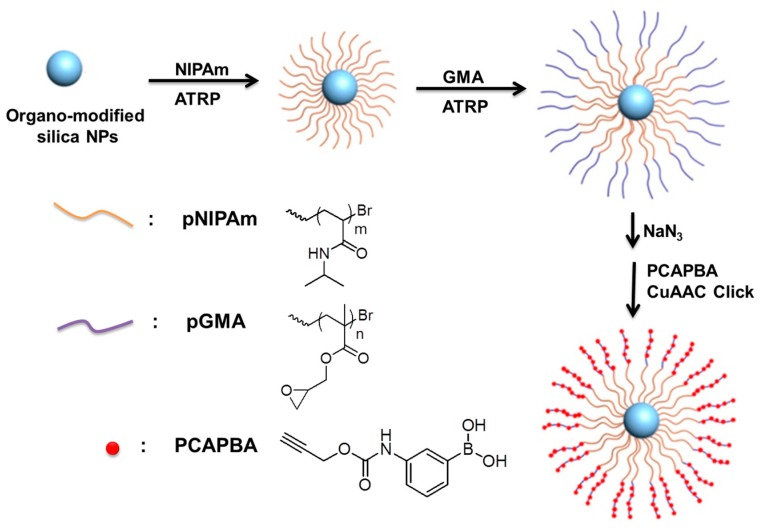
Preparation of boronic acid functional silica-based nanocomposites via the combination of surface-initiated ATRP and CuAAC click reaction. Adapted with permission from [31]. Copyright © 2017 by the American Chemical Society.

Polymer silica nanocomposites have been extensively studied in which silica-reinforced composites convey well of the optimized balance of strength/stiffness and toughness [30]. The applications of such materials span a variety of areas including, coatings, proton exchange membranes, encapsulation of organic light-emitting diodes, chemosensors and metal ion removal. CuAAC click chemistry has been well established in modification of silica particles and subsequent nanocomposite formation with polymer matrix. In an example, Ye and co-workers combined the atom transfer radical polymerization (ATRP) and click chemistry to prepare polymer nanocomposites containing silica NPs [31]. Surface-initiated ATRP of *N*-isopropylacrylamide and glycidyl methacrylate from silica NPs was conducted and the resulting polymer chain ends were transformed into azide functionalities. Afterwards, the alkyne group containing boronic acid was conjugated to the polymer brush-modified nanoparticle surface by employing Cu(I)-catalyzed CuAAC click reaction (Figure 3). These nanocomposites demonstrated high binding capacity towards glycoproteins of ovalbumin and horseradish peroxidase and provided pH- and temperature-responsive binding efficiency. Therefore, they are especially useful in bioseparation of important biomolecules and in other biomedical applications. 

Virtanen et al. designed an epoxy matrix-based nanocomposite with modified silica nanoparticles exhibiting dielectric properties [32]. The synthesis of nanocomposite included the azide functionalization of silica nanoparticles and subsequent coupling to alkyne-functionalized poly(glycidyl methacrylate), oligothiophene and ethynylferrocene species using CuAAC click reaction. The final product was obtained by using a bisphenol A-based epoxy resin and an aliphatic amine-based hardener. This material provided well dispersion of silica nanoparticles within the polymer matrix and allowed the study of effects of electroactive units on the dielectric breakdown strength.

The polyhedral oligomeric silsesquioxane (POSS) reagents are widely investigated as nanofillers, because their incorporation into the polymer matrix can drastically change the mechanical properties [33]. Functionalization of POSS derivatives with clickable groups can effectively be implemented and the resulting POSS derivative can be incorporated into the polymer scaffold via grafting, copolymerization or blending. POSS is a multi-functional reagent and this multi-functionality can be utilized in tuning of material properties. As an example, mono azide functional POSS was synthesized and used in fabrication of poly(*N*-isopropyl acrylamide)-based thermoresponsive nanocomposite hydrogels [34]. Thermally curable polybenzoxazine nanocomposite of multi-azide functional POSS [35], star and network polymers [36,37,38], linear polypeptide [39] and poly(HEMA-*co*-MMA)-based nanocomposites of mono-azide functional POSS [40] were also fabricated through CuAAC click methodology. 

Polymer/clay nanocomposites possessing unique physical, thermal and mechanical properties have gained a great deal of attention in both academia and industry [41]. Though the first reports of clay-based composites date back to the 1960s [42], Toyota’s report of a Nylon 6/clay nanocomposite [43] has accelerated the research on the topic. Clays are layered silicates of natural or synthetic minerals and display regular stacks of aluminosilicate sheets. The thickness of layers is about 1 nm and, upon unfolding, they provide high aspect ratio and high surface area. In terms of their roles as reinforcing fillers and to improve the nanocomposite properties, the ultimate choice is to completely disperse the clay plates in the polymer matrix [44]. To achieve this, the proper organic modification of clay is necessary and has utmost importance to effectively disperse the nanolayers in polymer host. Three mainly utilized methods of polymer/clay nanocomposite fabrication; solution exfoliation, melt intercalation and in situ intercalative polymerization [45,46,47,48,49,50,51] result in intercalated (inserting polymer chains into the layered space but keeping the periodic array of the clay platelets) or exfoliated (insertion of polymer chains by separating the clay sheets) structure [52,53]. 

Click chemistry-based approaches have provided a handy tool in the aforementioned methods of polymer/clay nanocomposite fabrication [44,54]. Tasdelen et al. have reported the first example of polymer/clay nanocomposite preparation by using CuAAC click reaction [55]. In the study, organo-modified montmorillonite (MMT) clay of bearing hydroxyl functionalities was decorated with azide functional groups and subsequently used in CuAAC click coupling with alkyne-functionalized polytetrahydrofuran. According to the characterization studies, the strategy provided good exfoliation of the clay in polymer matrix. The material provided improved thermal stability compared to the unmodified polymer. In a subsequent study of group, the azide decoration was utilized in intercalation of alkyne functionalized methacrylate monomer into the interlayer regions of montmorillonite [56]. Exfoliation of clay nanolayers was provided by in situ photo-initiated free radical polymerization of clay-supported monomer with methyl methacrylate (MMA). The efficient intercalation of monomer and exfoliated nanocomposite formation was demonstrated by X-ray diffraction spectroscopy (XRD, Rigaku D/max, Tokyo, Japan), transmission electron microscopy (TEM, FEI Tecnai G2 F20 S-Twin, Eindhoven, The Netherlands) and atomic force microscopy (AFM, NT-MDT Solver P47, Moscow, Russia). Thermal stability analysis of PMMA/MMT nanocomposites showed higher thermal stabilities of nanocomposites compared to that of pristine PMMA. Tasdelen recently reported the direct preparation of exfoliated polymer/clay nanocomposites by CuAAC click coupling of alkyne-functionalized poly(ε-caprolactone) and azide-modified montmorillonite [57].

Synthesis and performance study of sulfonated polytriazole- montmorillonite nanocomposites as proton exchange membranes of fuel cells was reported by Chang and co-workers [58]. Nanocomposites were fabricated by combining in situ polymerization and CuAAC click reaction. Differently from the azide functionalization, the clay was modified with alkyne groups via cationic exchange of Na^+^-MMT interlayered ions with quaternary ammonium and alkyne-bearing clay-modifying agent. The exfoliation of clay and nanocomposite formation was provided by in situ CuAAC click polymerization. The applied strategy allowed the well dispersion of clay layers and provided enhancements in thermal and mechanical properties, as well as methanol permeability, water retention, ion channel size, and ionic cluster distribution.

Bionanocomposite films of CuAAC click-functionalized halloysite nanotubes with chitosan and hydroxypropyl cellulose [59] and copper-free click functionalizable laponite/polyether nanocomposites were recently reported as nanoclay-reinforced polymeric biomaterials [60].

An exceedingly fast-growing research area is based on nanocomposites of graphene and its analogues: graphite, graphene oxide, carbon nanotubes, fullerenes, etc. Graphene is composed of two-dimensional sp^2^ carbon atoms arranged in a sheet, whereas graphite is a three-dimensional array of graphene layers. The oxidation of natural graphite with strong oxidation agents and exfoliation yields graphene oxide (GO) in which GO can be further deoxygenated to graphene. Fullerenes and nanotubes can be viewed as wrapped sections of graphene. Due to superior characteristics of graphene analogues such as electric, optoelectric, ferroelectric and piezoelectric properties, energy storage, electromagnetic interference shielding, photovoltaic properties and microwave absorption, these carbon-based materials are widely applied in polymer nanocomposite fabrication [61]. 

CuAAC click chemistry is a versatile tool for covalent functionalization of graphene and graphene oxide [62]. The chemistry can be implemented on solution-dispersed graphene/GO substrate [63] and the solvent chosen may have an impact on final material properties [64].

By taking advantage of CuAAC click reaction, Cao et al. functionalized the graphene oxide sheets with SEBS (styrene ethylene butylene styrene) copolymer [65]. The strategy included the azide modification of SEBS and alkyne modification of GO and subsequent click coupling of complementary groups to produce highly organo-soluble nanocomposites. SEBS-grafted GO was applied as reinforcing filler for polystyrene and resulted in 78% improvement in tensile strength and 73% increase in Young’s modulus. 

**Figure 4:**
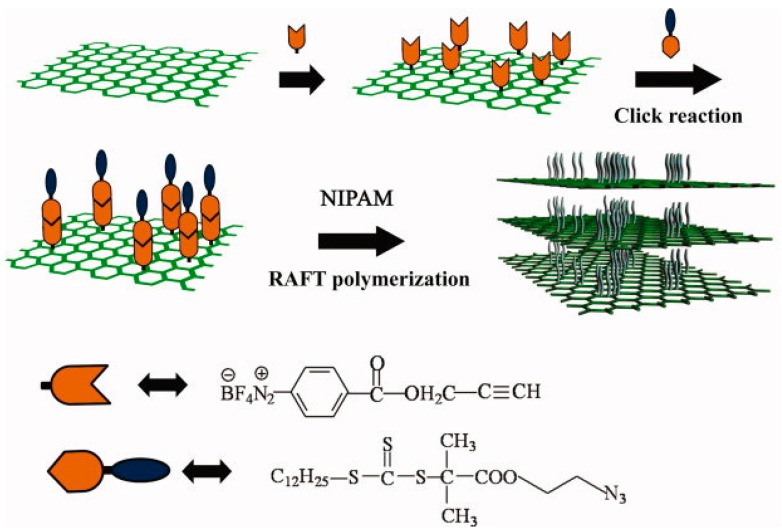
Fabrication of p(NIPAm)/reduced graphene oxide nanocomposites via CuAAC click reaction and surface-initiated RAFT polymerization. Adapted with permission from [66]. Copyright © 2011 by Wiley Periodicals, Inc.

Zhao and co-workers demonstrated the reversible addition-fragmentation chain-transfer (RAFT) functionalization of reduced GO by employing “grafting from” approach [66]. Polymer growth from GO was carried out by CuAAC click attachment of a chain transfer agent onto GO surface and subsequent RAFT polymerization of *N*-isopropylacrylamide (NIPAm) (Figure 4). A similar strategy was employed by Pan et al. to produce poly(*N*-isopropylacrylamide) (pNIPAm) functionalized graphene sheets [67]. Differently, polymer covalent attachment was maintained via a “grafting onto” approach as azide functional polymer was click coupled onto alkyne functional GO surface. Preparation of poly(*N*-(2-hydroxypropyl) methacrylamide)/GO nanocomposites via CuAAC click and grafting-onto strategy was also successfully implemented [68]. 

Poly(ε-caprolactone) (PCL)-functionalized GO was utilized in fabrication of polyurethane nanocomposites [69]. Azide-functionalized PCL was effectively anchored onto alkyne-decorated GO under CuAAC conditions. The PCL-modified GO demonstrated excellent compatibility with polyurethane (PU) matrix and provided increased mechanical and thermal properties, and thermal conductivity. Another polyurethane-based modified GO nanocomposite was reported by Cho and co-workers recently [70]. A novel strategy was employed as chemically stitched graphene oxide (GO) sheets were fabricated by covalent click coupling of bare azide and alkyne functional GOs. The PU nanocomposites of GO-click-GO sheets demonstrated improved breaking stress, modulus and photothermal properties than the conventional GO/PU nanocomposite. The CuAAC click-coupled exfoliation and nanocomposite formation by using azide-functionalized hyperbranched polyurethane (HBPU) and alkyne-grafted GO was recently reported [71]. The direct CuAAC-aided synthesis of nanocomposites by using thermally reduced graphene oxide-supported copper nanoparticles (TRGO-Cu_2_O) as catalyst system was successfully performed by Nia et al. [72]. The study of (TRGO-Cu_2_O) system directly embedded into the curing resin demonstrated superior performance in crosslinking, and the resulting nanocomposite exhibited enhanced mechanical and physical properties, as well as improved conductivity and thermal stability.

Bionanocomposites containing GO nanofiller are gaining importance as GO has become prominent in biotechnology fields, such as drug delivery [73], gene delivery [74] and photothermal cancer therapy [75]. Although the biocompatibility of graphene and graphene oxide has been debated [76], effective polymer modification may largely overcome the difficulties and facilitate advanced opportunities in both in vivo and in vitro biomedical applications. In this regard, polyethylene glycol (PEG)-functionalized nanographene oxide was synthesized via CuAAC click reaction and used in cancer cell targeting [77]. Recently, click chemistry-fabricated GO-based hydrogels [78] and GO/chitosan bionanocomposites [79] were also studied as potential biomaterials. 

Graphite-based polymer nanocomposites have attracted far less attention than the graphene or GO, this is probably due to the difficulties encountered in melt processing of graphite in dispersion and exfoliation and challenging thermodynamic and/or kinetic factors during conventional processing [80]. However, a successful CuAAC click chemistry-based fabrication strategy was facilitated to enhance the exfoliation of nanographite in poly(methyl methacrylate) matrix [81]. Improved material properties, such as flexibility, durability and carrier mobility allowed the material to be used in ammonia gas sensor application.

Interpenetrating polymer networks nanocomposites of multi-walled carbon nanotubes (MWCNTs) [82] and surface-confined polyaddition from tethered MWCNTs [83] have been recently demonstrated by using CuAAC click chemistry.

### 2.2. Polymer Nanocomposites via Diels–Alder Reactions

In most cases, the DA reactions well obey the click concept: [4+2] cycloaddition reactions between electron-rich dienes and electron-deficient dienophiles with high efficiency, mild reaction conditions, tolerance to diverse functional groups and most of the time no byproduct formation [84]. The DA reactions have been particularly utilized in synthetic organic chemistry as a convenient method of forming carbon–carbon bonds allowing good control over regio- and stereo-selectivity [85]. The concept has also been extended to carbon–hetero bond formations (such as C–N, C–O and C–S bonds) via hetero-Diels–Alder reaction. The DA reactions are thermoreversible under particular cases and this thermoreversibility (combination of forward reaction; DA cycloaddition and reverse reaction; cycloreversion or retro-Diels–Alder reaction) provides a handle for tuning the material properties [86].

The DA reactions possess unique advantages on macromolecular science, and provide versatile approaches, especially in combination with controlled polymerization methods, to access diverse macromolecular architectures [87]. The temperature-dependent reversibility of DA reaction also makes it a desirable candidate for fabrication of self-healing materials [75]. 

From the early examples [88] to diverse applications, the Diels–Alder-based strategies have been extensively utilized in polymer nanocomposite fabrication. The MWCNT-reinforced nanocomposites have attracted special attention since the surface of carbon nanotube can be a ready component in the DA reactions. The initial reports suggested that MWCNTs can effectively undergo cycloaddition reactions with electron-deficient tetrazine dienes [89]. The possible [4+2] cycloaddition of MWCNT surface is not limited to electron-deficient dienes and many other substrates have been utilized in polymer nanocomposite formation. For example, benzocyclobutene derivatives were employed as latent dienes that can undergo thermal ring opening at elevated temperature (>225 °C) to form o-quinodimethane radical intermediates [90]. The resulting biradicals can effectively couple with sp^2^ carbons of MWNTs through DA cycloaddition. This approach was also utilized to anchor nanotube surface with anionic initiators [91]. The covalent attachment of 4-hydroxyethyl benzocyclobuteneand 1-benzocyclobutene-1′-phenylethylene to nanotube surface was accomplished through Diels–Alder cycloaddition performed at 235 °C. Anionic polymerization from the nanotube was conducted to result in polystyrene and polyethylene oxide-grafted nanocomposites. The strategy was also successfully applied to surface-initiated ring opening polymerization of ε-caprolactone [92] and surface-initiated titanium-mediated coordination polymerizations of l-lactide [93]. A recent study of cyclobutene-based DA nanocomposite formation focused on the mechanistic study of nucleation and antinucleation effects of polymer grafted single-walled carbon nanotubes (SWCNT)s and MWCNTs on cyclic and linear poly(ε-caprolactone)s [94]. The MWCNTs can contribute DA reactions either as a dienophile or as a diene [95]. The maleimide group, a very popular activated dienophile for instance, was employed as a cycloaddition bridge between Fe_3_O_4_ nanoparticles and MWCNT surface [96]. 

Furan is another important cycloaddition substrate in DA reactions and, in the case of furan, MWCNT surface acts as dienophile. The furan-modified benzoxazine was directly implemented on the nanotube surfaces at 80 °C in dimethylformamide DA click reaction [97]. Subsequent polymer grafting from surface was carried out by using bisfuran benzoxazine and bismaleimide building blocks and employing DA cycloaddition polymerization. The polymer nanocomposite fabrication was performed via thermal curing of benzoxazine units at 180–240 °C. The thermoreversibility of DA process was investigated by heating the benzoxazine functionalized MWCNT at 160 °C for 3 h. The FT-IR analysis confirmed the loss of the absorptions of the organic moieties and hence proved the performance of the retro-DA reaction. The self-healing ability of a MWCNT/(poly(ester-urethane)-poly(ε-caprolactone)) nanocomposite, fabricated via DA reaction, was investigated for the first time to heal the macroscopic failure locally by Joule effect [98]. The studies showed that the confined temperature increase arising from Joule effect can heal the damage near the damaged site by the help of retro-Diels–Alder reaction (rDA). The proposed mechanism is based on the repair driven by the accumulation of rDA-generated low molecular weight oligomers at crack region and further DA curing at the crack site. Semiconductive nanocomposites of MWCNTs with poly(3-hydroxybutyrate-*co*-3-hydroxyhexanoate) were also prepared via DA reaction of furan end-group-modified polymer with nanotube surface [99]. The resulting material displayed good dispersion of MWCNTs in polymer matrix and almost conductive nanocomposites were obtained in the case of 1.2 wt % of pol-MWCNTs.

**Figure 5:**
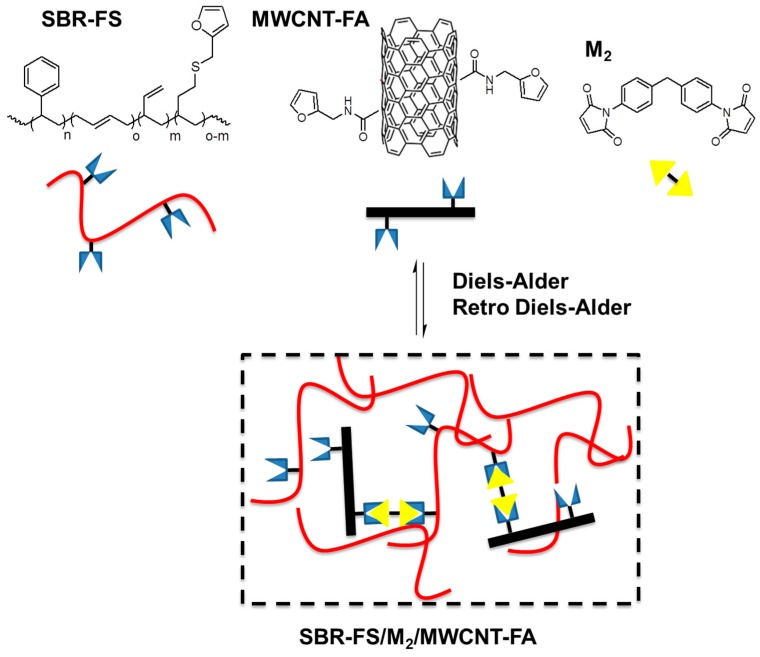
Synthesis of covalently bonded and reversibly cross-linked rubber nanocomposites via Diels–Alder reaction. Adapted with permission from [103]. Copyright © 2016 by WILEY-VCH Verlag GmbH & Co.

The functionalization of MWCNTs through the DA click reactions brings an important advantage in which the nanotube surface can be directly implemented without the need of any initial destructive surface treatment. Contrarily, the conventional methods may physically damage the nanotube surface or shorten the nanotube length with deterioration in mechanical properties [100,101]. The minimum destruction of nanotube side walls associated with the minimum sp^2^ deterioration may increase the performance of fabricated nanocomposites especially in sensor applications [102]. The thermoreversible nature of DA reaction was recently utilized in fabrication of self-healing rubber nanocomposites of MWCNTs with styrene-butadiene rubber (SBR) [103]. The nanocomposites were obtained by DA reaction of furan-modified MWCNTs and furan-modified SBR with a bismaleimide functional crosslinker (Figure 5). The mechanical studies demonstrated that the loading of furan functionalized MWCNT in 5 wt % provided 2–3-fold increase in the Young’s modulus and toughness. The self-healing tests, performed by the comparison of fracture strains of original specimens and mechanically torn and reunited samples, exhibited efficient healing of nanocomposites upon mild thermal treatment.

The function and utility of inorganic NPs are greatly affected by surface properties and are dominated by interfacial energies which most of the time requires elaborate chemical modification. The DA reaction, as an efficient click methodology, provides fine tuning of fabrication methodologies and material properties in polymer nanocomposite fabrication. A series of nanoparticles including gold, silver, iron oxide, ceramic and silica nanoparticles can be used as reinforcing fillers in polymer nanocomposites, transferring their unique properties to fabricated material. Gold nanoparticles (Au NPs) for example, have low toxicity [104] and high biocompatibility and thus are widely used in biomaterial fabrication. Gabilondo and co-workers have employed Au NPs in bionanocomposite hydrogel fabrication with the aid of DA reaction [105,106]. In the approach, thiol binding ability of gold nanoparticles was utilized for straightforward modification of NP surface with maleimide groups allowing DA coupling with furan-modified gelatin and chitosan. The maleimide functionalization of Au NPs was also utilized by Liu et al. in DA-mediated preparation of homogeneous and electronically conducting polymer nanocomposites [107].

**Figure 6:**
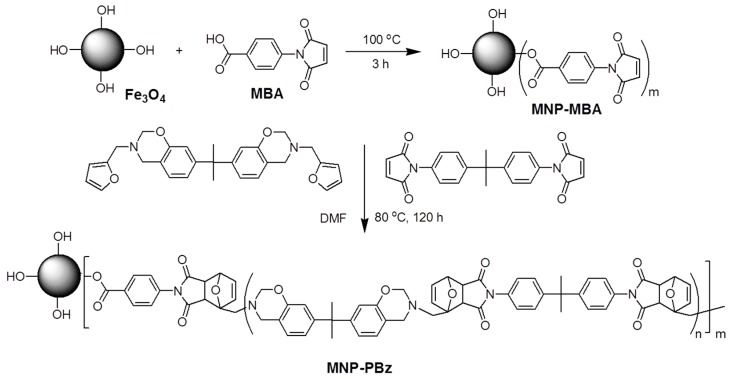
Preparation of Fe_3_O_4_ NPs/polybenzoxazine grafts via DA reaction. Adapted with permission from [109]. Copyright © 2016 by WILEY-VCH Verlag GmbH & Co.

Bionanocomposite hydrogels of maleimide-modified Ag NPs with furan-functionalized gelation through DA reaction was demonstrated by Gabilondo group [108]. These materials offered low toxicity, higher storage modulus and sustained drug release, making them ideal candidates for various biomedical applications. In a recent study, the polybenzoxazine nanocomposites reinforced with Fe_3_O_4_ NPs were prepared through DA click reaction [109]. The Fe_3_O_4_ NPs display superparamagnetism and this has received attention especially in biomolecule separation, magnetic resonance (MR) imaging, targeted drug delivery and toxic chemical and metal ion removal. The strategy was based on maleimide functionalization of Fe_3_O_4_ NPs and subsequent DA click reaction with bisfuran-modified benzoxazine and bismaleimide precursors (Figure 6). Upon thermal treatment of benzoxazine moieties, the desired nanocomposites were obtained with increased mechanical properties, high glass transition temperatures and magnetically responsive property.

The polymer growth from nanoparticle surface (graft from) is advantageous for the preparation of nanoparticle-based polymer nanocomposites, since in the DA functionalization of NP surface with already prepared polymers (grafting onto), steric factors could be a problem and diminish the DA coupling efficiency [110]. The surface-initiated polymerization from nanoparticles provides a handy tool to incorporate DA-reactive precursors in multiple quantities along the polymer backbone. This was employed by Engel et al. to prepare self-healing nanocomposites of silica NPs with combination of surface-initiated ATRP (SI-ATRP) and DA reaction [111]. First, an ATRP initiator was attached onto the nanoparticle surface, then SI-ATRP was conducted by using butyl methacrylate along with maleimide- or furan-functionalized methacrylate monomers. Finally, upon heat treatment, thermally-healable nanocomposites were obtained via Diels–Alder click reaction. In a subsequent study of the same group, the role of polymer matrix in self-healing abilities of silica NPs nanocomposites was investigated [112]. The thermally self-healing and mechanically enhanced nanocomposites of multi-methacrylated POSS with furfurylamine [113] and multi furan-grafted POSS with bismaleimide [114] were recently reported as elaborate examples of DA-based nanofabrication. Gabilondo group has extended the surface maleimide functionalization to TiO_2_ NPs by using a catechol-based maleimide linker [115]. Catechol derivatives possess excellent binding ability toward various inorganic substrates and allow incorporation of numerous functional groups onto the material surface [116,117]. In the study, maleimide-modified TiO_2_ NPs allowed efficient preparation of bionanocomposite hydrogels by crosslinking with furan-modified gelatin. In addition to catechol-based functionalization of nanoparticle surfaces with DA adducts, silane derivatives can also be used as surface modifiers. This strategy was recently used by Yang et al. to anchor CaCu_3_Ti_4_O_12_ nanoparticle surface with maleimide moieties [118]. Flexible and thermally healable nanocomposites were obtained by DA crosslinking of modified NPs with dynamic Diels–Alder adducts in the recoverable motion sensor application. 

In a rare example of employing DA reaction in the preparation of nanocomposites is gelatin-based fully-renewable bionanocomposites including CNC as nanofiller [119]. 

The conducting polymer-based nanocomposites (polyanilines (PANI), polypyrroles (PPy), poly(3,4-ethylenedioxythiophene) (PEDOT), etc.) exhibited additional electrical conductivity with high mechanical properties and largely studied in flexible electronics, supercapacitors and related applications. Graphene and GO reinforcers prove versatility and efficiency in these applications because of their high surface conductivity and electrochemical stability [120]. The DA click reaction provides a handy tool both in fabrication and modification of such electro-active nanocomposites [121]. In the case of flexible electronics of elastomers with graphene and GO, DA-based self-healing upon fracture is a means of achieving durability and stability [122].

### 2.3. Polymer Nanocomposites via Thiol-X Reactions

The reactions of thiols with alkenes and alkynes can be collected conceptually under “thiol-X reactions” in which these reactions have been utilized in numerous applications of macromolecular science and material research [123]. The thiol-X reactions represent a very versatile group of synthetic methodologies that share most of “click” properties: mild reaction conditions, rapid reaction kinetics with high product yields, chemoselectivity (the anti-Markovnikov addition products in case of ene and yne substrates) and few to none side product formation [124]. From the materials science aspect, thiol-X chemistries generally provide simpler and more benign synthetic procedures and, under proper conditions, they go more rapidly than various other click reactions [125]. Although a wide range of thiol-X chemistries including radical thiol-ene, thiol-yne, nucleophilic thiol-ene (or thiol Michael addition), thiol-halogen, thiol-epoxy and thiol-isocyanate reactions are referred to as “thiol-click chemistry” [126,127], much of the research on polymer nanocomposite fabrication is based on radical thiol-ene, nucleophilic thiol-ene and thiol-yne reactions.

Recently, radical thiol-ene reaction was employed in preparation of CNCs with polybutadiene rubber [128]. In the study, CNCs were surface-modified with thiol-ene clickable alkene groups and subsequently used in UV-light-initiated photocrosslinking of multi-alkene-containing polybutadiene. It was shown that high CNCs loading results in tightly packed self-assembled and aligned domains, thus allowing practical routes for biomimetic composites. In another study, CNCs were functionalized with thiol units and thiol-ene click coupled with natural rubber [129]. Compared to nanocomposites of natural rubber with unmodified CNCs, the thiol-ene-fabricated natural rubber/CNCs nanocomposites showed increased tensile strength, strain-to-failure and work-of-fracture.

In an elegant strategy, Rowan and co-workers developed nanocomposite films of CNCs embedded in poly(vinyl acetate) (PVA) matrix by mimicking water-enhanced mechanical gradient properties of the squid beak [129]. The films were fabricated through radical thiol-ene reaction of alkene-modified reinforcer with a tetra-thiol functional crosslinker. It was shown that, by tuning the degree of crosslinking and photo-irradiation exposure time of different sections of the film, a gradient degree of covalent crosslinking could be achieved. This gradient crosslinking resulted in significant mechanical contrast along the film in hydrated state, due to the “switches off” of the noncovalent CNCs interactions. Schyrr et al. reported the preparation of CNCs/poly(vinyl alcohol) nanocomposite scaffolds via nucleophilic thiol-Michael addition reaction [130]. Acrylate-modified cotton-derived CNCs and PVA were dip-coated and dried on glass substrates and the acrylate groups were allowed to immobilize various thiol probes. 

Thiol-ene reactions prove to be convenient routes to prepare ceramic/polymer nanocomposites [131,132,133], as well as clay-based systems. A much sought strategy of polymer nanocomposite fabrication is based on acrylate-based photopolymerization techniques. However, some intrinsic drawbacks, such as shrinkage during polymerization, reduced gas barrier properties, abrasion and low impact resistance could often be encountered in these systems [134]. Thiol-ene photopolymerization-based network formation is a promising alternative strategy in which step-growth reaction mechanism of thiol-ene reaction may induce more homogeneous network formation and reduce the shrinkage during photopolymerization [135,136,137].

The photoinitiated thiol-ene concept was recently utilized in preparation of clay-reinforced transparent barrier nanocomposite films [138]. The incorporation of inorganic clay into the nanocomposite effectively enhanced the mechanical properties; however, in the case of >1% clay composition, worsening in optical transparency was observed. Guymon and co-workers extensively investigated the nanocomposite physical properties with respect to reaction behavior [139,140]. Their studies revealed that photopolymerization behavior and final composite properties are largely influenced by the organo-modification of clay surface with appropriate functional groups and degree of clay exfoliation. Increased reaction rates and conversions were obtained in the case of using acrylate- or thiol-modified organoclay systems compared to unmodified clay nanocomposites. Use of thiolated organoclay reduced the polymerization-induced shrinkage of the network to the comparable neat organoclay system. The degree of clay exfoliation can also be controlled by introducing either thiol or alkene groups onto the clay surfaces.

**Figure 7:**
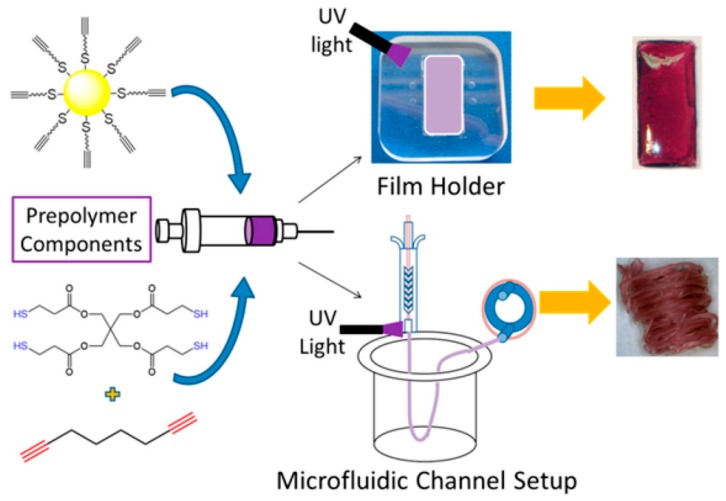
Fabrication of film and fiber nanocomposites of Au NPs via thiol click chemistry. Adapted with permission from [141]. Copyright © 2013 by the American Chemical Society.

An intriguing example of thiol-ene chemistry is the fabrication of color-tunable film and fiber nanocomposites reported by Boyd et al. [141]. In particular, alkene and alkyne-functionalized gold (Au) NPs were combined with multifunctional alkene/alkyne precursors and with a multifunctional thiol crosslinker to give corresponding films and fibers via thiol-ene and thiol-yne click reactions (Figure 7). The network formation was established in less than 30 m in films and was occurred in ca. 1 h for fibers, which is a drastic change in fabrication time compared to a previously reported process [142], which takes 3 weeks to incorporate Au NPs into the thiol-ene films. The report demonstrated that the optical and mechanical properties of nanocomposites could be tuned by using appropriate functional ligand, as well as by changing nanoparticle concentration. In a subsequent study, the group reported on the surface-enhanced Raman spectroscopy probe efficiency of thiol-yne nanocomposite films and fibers of Au NPs [143]. Poly(ethylene glycol)-based hydrogels containing Au NPs as nanofillers were reported by Ren et al. [144]. Silicon surface-adsorbed Au NPs were transferred to thiol-ene functionalized soft hydrogels via a nanocontact deprinting method. It was estimated that the transfer efficiency was 83% based on scanning electron microscope (SEM) analysis. The Au NPs immobilized hydrogels were shown to allow controlled cell adhesion and spreading of fibroblast L-929 cells.

Polymer nanocomposite coatings have been thoroughly investigated in which their applications span from imaging, anticorrosion, marine, smart packaging materials, automotive industry to high technology applications [145,146,147]. Nanocomposite coatings can provide high-efficiency gas barrier to the substrate foil [148] and they can provide superior anti-scratch and anti-corrosion properties. Recently, thiol-ene click reaction was utilized in preparation of highly hydrophobic and oleophobic fluorinated coatings with the aid of hydrophobic-fumed nanosilica particles [149]. The addition of silica nanoparticles provided enhancement in the mechanical properties of the coatings. The materials also displayed high thermal stability and high solvent resistance. In another application, silica NPs-reinforced and surface-attached pNIPAm hydrogels were fabricated by thiol-ene click chemistry [150]. The thiol-ene approach provided well-controlled and tailored architecture of film formation on various thickness supports. In addition to silica NPs, POSS-based reinforcers are also common in thiol-ene- and thiol-epoxy-based coating applications [151,152,153]. POSS endows high reactivity resulting from multifunctional nature and high compatibility with polymer matrix by providing enhanced thermal-mechanical properties and high oxidation-chemical resistance [154,155].

Porous silicon-based nanocomposites have received attention in biomedical applications because these nanoparticles offer several desired features: large surface area, adjustable pore size and pore volume, high biocompatibility, easy surface modification and high capacity loading of related compounds, such as drug molecules [156,157]. Since thiols are widely encountered in biological systems [158], thiol click chemistry provides versatility in designing bionanocomposite biomaterials [159]. In applications of nanoparticle-reinforced materials, thiol-ene nanocomposites of superparamagnetic iron oxide nanocrystals with poly(ethylene oxide) [160], silver nanoparticles [161] and polymer/quantum dot nanocomposite thin films for optical and optoelectronic applications [162] were recently reported.

**Figure 8:**
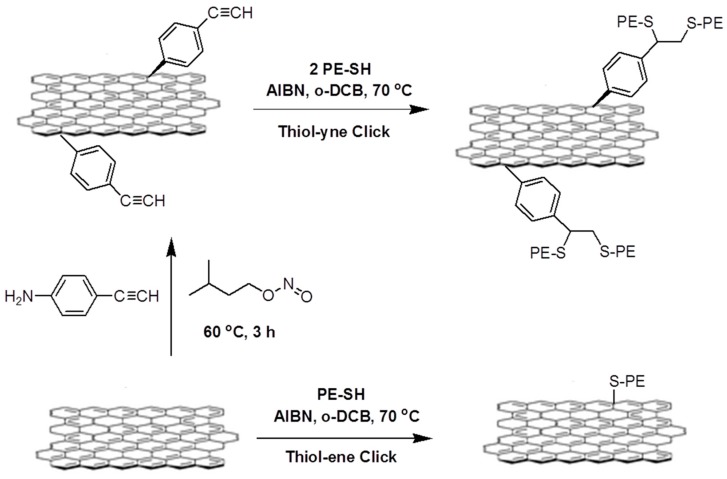
Thiol-ene- and thiol-yne-mediated polymer grafting from graphene surface. Adapted with permission from [170]. Copyright © 2013 by The Royal Society of Chemistry.

Thiol-ene click reactions are among the most applied chemical tools for graphene/GO-based polymer nanocomposite fabrication [163,164]. The surface of graphene and GO acts as a direct ene substrate in radical thiol-ene reaction and allows very efficient covalent functionalization that can be far successful beyond the other click-based methodologies [165]. The “on demand” nature of radical thiol-ene reaction (activation by UV-light) is also a practical opportunity especially in fabrication of graphene/GO-based nanocomposite coatings [166,167,168,169]. Although the chemical modification of graphene surface with alkynes may cause deterioration in sp^2^ carbon array, it allows double-branched modification through thiol-yne reaction (Figure 8) [170]. Similar to Diels–Alder functionalization of MWCNTs, the pristine nanotube surface can act as an ‘ene’ component in radical thiol-ene reaction. This property can be exploited in, for example, direct formation of MWCNTs nanocomposites [171]. Thiol-ene reaction also provides efficient conjugation of small molecules onto conducting polymer-coated MWCNTs in which these nanocomposites can be beneficial in electro-analytic applications [172,173,174].

## 3. General Overview and Future Perspectives

The click chemistry-based concepts in polymer nanocomposite fabrication draw attention because of the high efficiencies of these coupling reactions that allow high reaction rates and reaction yields, relatively mild reaction conditions, orthogonality and experimental simplicity. Especially in the case of taking the advantage of placing reactive groups at well-defined positions of polymer precursors (such as end-group functional telechelic polymers), it might be possible to better control the degree of crosslinking and thus achieve more precise control on the network structure. This is especially desirable for reproduction of the material and to attain improved structure–property relationship. Versatilities of click reactions can not only be exploited in the synthesis of polymer nanocomposites; instead, they can provide a vide scope to further functionalize fabricated materials with various accessories for various applications. For example, in biomedical applications of nanocomposite materials, conjugation of drugs, enzymes, proteins or other related bioagents is highly needed to impart expected function. Highly efficient and bioorthogonal click methodologies may provide efficient functionalization. Click reactions possess different reaction conditions such as metal catalyst operational or catalyst-free reaction coordinates, thermally driven or UV-triggered activations, reversible or irreversible reaction mechanisms, etc. These diverse opportunities could be beneficial while deciding the optimum material manufacturing process. Table 1 summarizes the prominent advantages and limitations of common click reactions in polymer nanocomposite fabrication. 

The applications of click methodologies in polymer nanocomposite fabrications is not limited to the above-mentioned common click reactions. For example, hydrazone [175,176] and oxime-based [177] carbonyl chemistries have also found applications in polymer nanocomposite-based biomaterials. A new-born and fast-growing click reaction of sulfur(VI) fluoride exchange (SuFEx), pioneered by K.B. Sharpless and colleagues, could provide new insights into polymer nanocomposite design and manufacture. The inverse-electron-demand Diels–Alder reaction of tetrazines and strained alkene dienophiles [178] and 2-cyanobenzothiazole-based [179] click concepts might open new research areas in functional nanocomposite materials. 

## 4. Conclusions

As focused in this review article, a large spectrum of research activity has been dedicated to utilization of click chemistry methodologies toward the preparation of polymer nanocomposites. Click reactions have gained acceptance as robust, efficient and reliable procedures and could serve broad opportunities in design, fabrication and application of various functional materials. Despite the fact that employing click methodologies on polymer nanocomposite fabrication is a relatively new concept and much of the research is in the last decade, much further advancements can be anticipated in applications of these fruitful chemical tools.

## Figures and Tables

**Table 1:** Main advantages and associated limitations of commonly employed click reactions in polymer nanocomposite fabrication.

Click Reaction	Advantages	Limitations	Comments
CuAAC click reaction	High selectivity, rapid and quantitative transformations, tolerance to diverse organic solvents and water, stability, orthogonality.	Need to use (toxic) metal catalyst. Removal of the catalyst.	Particularly suitable for effective coupling of nanofillers and matrices. Provides improvement in interfacial compatibility.
Metal-free click reaction	Selectivity, high reactivity, biocompatibility and stability.	Substrates such as strained cyclooctynes are not so common and expensive materials.	Suitable if toxic metal catalyst is an issue, especially in biomedical applications.
Diels–Alder reaction	Activation through heating (could be beneficial in certain cases). Most of the time no byproduct formation. Reversibility of the reaction.	Heating requirement (could be a problem in certain cases), relatively prolonged reactions times.	Nanofiller surface can act as a substrate which resolves the destructive surface chemical treatments. Reversible nature is useful in self-healing materials.
Thiol-ene and thiol-yne reactions	High efficiency, high conversions, UV or heat-triggered activation.	Thiols are prone to many side reactions and have low self-stability. Especially the volatile thiols have disagreeable odors.	Reaction mechanism may induce more homogeneous network formation and reduces the network shrinkage. UV-triggered nature might be useful in coating applications.

## References

[1] Alexandre M., Dubois P. (2000). Polymer-layered silicate nanocomposites: Preparation, properties and uses of a new class of materials. Mater. Sci. Eng. R.

[2] Hussain F., Hojjati M., Okamoto M., Gorga R.E. (2006). Polymer-matrix nanocomposites, processing, manufacturing, and application: An overview. J. Compos. Mater..

[3] Sahoo N.G., Rana S., Cho J.W., Li L., Chan S.H. (2010). Polymer nanocomposites based on functionalized carbon nanotubes. Prog. Polym. Sci..

[4] Liu T., Burger C., Chu B. (2003). Nanofabrication in polymer matrices. Prog. Polym. Sci..

[5] Anandhan S., Bandyopadhyay S. (2011). Polymer nanocomposites: From synthesis to applications. Nanocomposites and Polymers with Analytical Methods.

[6] Kolb H.C., Finn M., Sharpless K.B. (2001). Click chemistry: Diverse chemical function from a few good reactions. Angew. Chem. Int. Ed..

[7] Moses J.E., Moorhouse A.D. (2007). The growing applications of click chemistry. Chem. Soc. Rev..

[8] Tron G.C., Pirali T., Billington R.A., Canonico P.L., Sorba G., Genazzani A.A. (2008). Click chemistry reactions in medicinal chemistry: Applications of the 1,3-dipolar cycloaddition between azides and alkynes. Med. Res. Rev..

[9] Binder W.H., Sachsenhofer R. (2008). ‘Click’ chemistry in polymer and material science: An update. Macromol. Rapid Commun..

[10] Sumerlin B.S., Vogt A.P. (2010). Macromolecular engineering through click chemistry and other efficient transformations. Macromolecules.

[11] Meldal M., Tornøe C.W. (2008). Cu-catalyzed azide–alkyne cycloaddition. Chem. Rev..

[12] Tasdelen M.A., Kiskan B., Yagci Y. (2016). Externally stimulated click reactions for macromolecular syntheses. Prog. Polym. Sci..

[13] Azeez A.A., Rhee K.Y., Park S.J., Hui D. (2013). Epoxy clay nanocomposites–processing, properties and applications: A review. Compos. Part B.

[14] Njuguna J., Pielichowski K., Alcock J.R. (2007). Epoxy-based fibre reinforced nanocomposites. Adv. Eng. Mater..

[15] Wei J., Vo T., Inam F. (2015). Epoxy/graphene nanocomposites–processing and properties: A review. RSC Adv..

[16] Rostovtsev V.V., Green L.G., Fokin V.V., Sharpless K.B. (2002). A stepwise huisgen cycloaddition process: Copper (I)-catalyzed regioselective “ligation” of azides and terminal alkynes. Angew. Chem. Int. Ed..

[17] Tornøe C.W., Christensen C., Meldal M. (2002). Peptidotriazoles on solid phase:[1,2,3]-triazoles by regiospecific copper (I)-catalyzed 1,3-dipolar cycloadditions of terminal alkynes to azides. J. Org. Chem..

[18] Baskin J.M., Prescher J.A., Laughlin S.T., Agard N.J., Chang P.V., Miller I.A., Lo A., Codelli J.A., Bertozzi C.R. (2007). Copper-free click chemistry for dynamic In Vivo imaging. Proc. Natl. Acad. Sci. USA.

[19] Chen J., Lin N., Huang J., Dufresne A. (2015). Highly alkynyl-functionalization of cellulose nanocrystals and advanced nanocomposites thereof via click chemistry. Polym. Chem..

[20] Liu H., Chung H. (2016). Self-healing properties of lignin-containing nanocomposite: Synthesis of lignin-graft-poly (5-acetylaminopentyl acrylate) via raft and click chemistry. Macromolecules.

[21] Viswanathan V., Laha T., Balani K., Agarwal A., Seal S. (2006). Challenges and advances in nanocomposite processing techniques. Mater. Sci. Eng..

[22] Kuchibhatla S., Karakoti A.S., Bera D., Seal S. (2007). One dimensional nanostructured materials. Prog. Mater. Sci..

[23] Tao P., Viswanath A., Schadler L.S., Benicewicz B.C., Siegel R.W. (2011). Preparation and optical properties of indium tin oxide/epoxy nanocomposites with polyglycidyl methacrylate grafted nanoparticles. ACS Appl. Mater. Interfaces.

[24] Tchoul M.N., Fillery S.P., Koerner H., Drummy L.F., Oyerokun F.T., Mirau P.A., Durstock M.F., Vaia R.A. (2010). Assemblies of titanium dioxide-polystyrene hybrid nanoparticles for dielectric applications. Chem. Mater..

[25] Zhang X., Kim J.S., Kwon Y. (2017). Synthesis and thermal analysis of nano-aluminum/fluorinated polyurethane elastomeric composites for structural energetics. J. Nanosci. Nanotechnol..

[26] Lakouraj M.M., Hasanzadeh F., Zare E.N. (2014). Nanogel and super-paramagnetic nanocomposite of thiacalix[4]arene functionalized chitosan: Synthesis, characterization and heavy metal sorption. Iran. Polym. J..

[27] Tian Y., Shi W., Luo J., Ma F., Mi H., Lei Y. (2013). Carbazole-based conjugated polymer covalently coated fe3o4 nanoparticle as efficient and reversible hg^2+^ optical probe. J. Polym. Sci. Part A.

[28] Kantheti S., Narayan R., Raju K.V. (2015). Pyrene-anchored ZnO nanoparticles through click reaction for the development of antimicrobial and fluorescent polyurethane nanocomposite. Polym. Int..

[29] Oz E., Uyar T., Esen H., Tasdelen M.A. (2017). Simultaneous photoinduced electron transfer and photoinduced cuaac processes for antibacterial thermosets. Prog. Org. Coat..

[30] Zou H., Wu S., Shen J. (2008). Polymer/silica nanocomposites: Preparation, characterization, properties, and applications. Chem. Rev..

[31] Jiang L., Messing M.E., Ye L. (2017). Temperature and ph dual-responsive core-brush nanocomposite for enrichment of glycoproteins. ACS Appl. Mater. Interfaces.

[32] Virtanen S., Krentz T.M., Nelson J.K., Schadler L.S., Bell M., Benicewicz B., Hillborg H., Zhao S. (2014). Dielectric breakdown strength of epoxy bimodal-polymer-brush-grafted core functionalized silica nanocomposites. IEEE Trans. Dielectr. Electr. Insul..

[33] Kuo S.-W., Chang F.-C. (2011). Poss related polymer nanocomposites. Prog. Polym. Sci..

[34] Wang L., Zeng K., Zheng S. (2011). Hepta (3,3,3-trifluoropropyl) polyhedral oligomeric silsesquioxane-capped poly(*N*-isopropylacrylamide) telechelics: Synthesis and behavior of physical hydrogels. ACS Appl. Mater. Interfaces.

[35] Wu Y.-C., Kuo S.-W. (2010). Synthesis and characterization of polyhedral oligomeric silsesquioxane (poss) with multifunctional benzoxazine groups through click chemistry. Polymer.

[36] Arslan I., Tasdelen M.A. (2016). POSS-based hybrid thermosets via photoinduced copper-catalyzed azide-alkyne cycloaddition click chemistry. Des. Monomers Polym..

[37] Tinmaz H.B., Arslan I., Tasdelen M.A. (2015). Star polymers by photoinduced copper-catalyzed azide-alkyne cycloaddition click chemistry. J. Polym. Sci. Part A.

[38] Doganci E., Tasdelen M.A., Yilmaz F. (2015). Synthesis of miktoarm star-shaped polymers with poss core via a combination of cuaac click chemistry, atrp, and rop techniques. Macromol. Chem. Phys..

[39] Lin Y.C., Kuo S.W. (2011). Self-assembly and secondary structures of linear polypeptides tethered to polyhedral oligomeric silsesquioxane nanoparticles through click chemistry. J. Polym. Sci. Part A.

[40] Islam M., Bach L.G., Park J.M., Hong S.S., Lim K.T. (2013). Synthesis and characterization of poly (hema-*co*-mma)-g-poss nanocomposites by combination of reversible addition fragmentation chain transfer polymerization and click chemistry. J. Appl. Polym. Sci..

[41] Nguyen Q.T., Baird D.G. (2006). Preparation of polymer–clay nanocomposites and their properties. Adv. Polym. Technol..

[42] Solomon D.H., Loft B.C. (1968). Reactions catalyzed by minerals. Part III. The mechanism of spontaneous interlamellar polymerizations in aluminosilicates. J. Appl. Polym. Sci..

[43] Kojima Y., Usuki A., Kawasumi M., Okada A., Fukushima Y., Kurauchi T., Kamigaito O. (1993). Mechanical properties of nylon 6-clay hybrid. J. Mater. Res..

[44] Tasdelen M.A., Kreutzer J., Yagci Y. (2010). In Situ synthesis of polymer/clay nanocomposites by living and controlled/living polymerization. Macromol. Chem. Phys..

[45] Altinkok C., Uyar T., Tasdelen M.A., Yagci Y. (2011). In Situ synthesis of polymer/clay nanocomposites by type ii photoinitiated free radical polymerization. J. Polym. Sci. Part A.

[46] Ozkose U.U., Altinkok C., Yilmaz O., Alpturk O., Tasdelen M.A. (2017). In-Situ preparation of poly (2-ethyl-2-oxazoline)/clay nanocomposites via living cationic ring-opening polymerization. Eur. Polym. J..

[47] Oral A., Tasdelen M.A., Demirel A.L., Yagci Y. (2009). Poly(cyclohexene oxide)/clay nanocomposites by photoinitiated cationic polymerization via activated monomer mechanism. J. Polym. Sci. Part A.

[48] Yenice Z., Tasdelen M.A., Oral A., Guler C., Yagci Y. (2009). Poly(styrene-b-tetrahydrofuran)/clay nanocomposites by mechanistic transformation. J. Polym. Sci. Part A.

[49] Dizman C., Ates S., Uyar T., Tasdelen M.A., Torun L., Yagci Y. (2011). Polysulfone/clay nanocomposites by In Situ photoinduced crosslinking polymerization. Macromol. Mater. Eng..

[50] Karamane M., Raihane M., Tasdelen M.A., Uyar T., Lahcini M., Ilsouk M., Yagci Y. (2017). Preparation of fluorinated methacrylate/clay nanocomposite via in-situ polymerization: Characterization, structure, and properties. J. Polym. Sci. Part A.

[51] Demir K.D., Tasdelen M.A., Uyar T., Kawaguchi A.W., Sudo A., Endo T., Yagci Y. (2011). Synthesis of polybenzoxazine/clay nanocomposites by in situ thermal ring-opening polymerization using intercalated monomer. J. Polym. Sci. Part A.

[52] Ray S.S., Okamoto M. (2003). Polymer/layered silicate nanocomposites: A review from preparation to processing. Prog. Polym. Sci..

[53] Pavlidou S., Papaspyrides C. (2008). A review on polymer–layered silicate nanocomposites. Prog. Polym. Sci..

[54] Aydin M., Tasdelen M.A., Uyar T., Jockusch S., Turro N.J., Yagci Y. (2013). Polystyrene/clay nanocomposites by atom transfer radical nitroxide coupling chemistry. J. Polym. Sci. Part A.

[55] Tasdelen M.A., Van Camp W., Goethals E., Dubois P., Du Prez F., Yagci Y. (2008). Polytetrahydrofuran/clay nanocomposites by in situ polymerization and “click” chemistry processes. Macromolecules.

[56] Oral A., Tasdelen M.A., Demirel A.L., Yagci Y. (2009). Poly(methyl methacrylate)/clay nanocomposites by photoinitiated free radical polymerization using intercalated monomer. Polymer.

[57] Tasdelen M.A. (2011). Poly(epsilon-caprolactone)/clay nanocomposites via “click” chemistry. Eur. Polym. J..

[58] Huang Y.-J., Ye Y.-S., Syu Y.-J., Hwang B.-J., Chang F.-C. (2012). Synthesis and characterization of sulfonated polytriazole-clay proton exchange membrane by in situ polymerization and click reaction for direct methanol fuel cells. J. Power Sources.

[59] Arcudi F., Cavallaro G., Lazzara G., Massaro M., Milioto S., Noto R., Riela S. (2014). Selective functionalization of halloysite cavity by click reaction: Structured filler for enhancing mechanical properties of bionanocomposite films. J. Phys. Chem. C.

[60] Topuz F., Bartneck M., Pan Y., Tacke F. (2017). One-step fabrication of biocompatible multifaceted nanocomposite gels and nanolayers. Biomacromolecules.

[61] Hu K., Kulkarni D.D., Choi I., Tsukruk V.V. (2014). Graphene-polymer nanocomposites for structural and functional applications. Prog. Polym. Sci..

[62] Segura J.L., Salavagione H.J. (2013). Graphene in copper catalyzed azide-alkyne cycloaddition reactions: Evolution from [60]fullerene and carbon nanotubes strategies. Curr. Org. Chem..

[63] Jin Z., McNicholas T.P., Shih C.-J., Wang Q.H., Paulus G.L., Hilmer A.J., Shimizu S., Strano M.S. (2011). Click chemistry on solution-dispersed graphene and monolayer cvd graphene. Chem. Mater..

[64] Castelaín M., Martínez G., Merino P., Martín-Gago J.Á., Segura J.L., Ellis G., Salavagione H.J. (2012). Graphene functionalisation with a conjugated poly (fluorene) by click coupling: Striking electronic properties in solution. Chem. Eur. J..

[65] Cao Y., Lai Z., Feng J., Wu P. (2011). Graphene oxide sheets covalently functionalized with block copolymers via click chemistry as reinforcing fillers. J. Mater. Chem..

[66] Yang Y., Song X., Yuan L., Li M., Liu J., Ji R., Zhao H. (2012). Synthesis of pnipam polymer brushes on reduced graphene oxide based on click chemistry and raft polymerization. J. Polym. Sci. Part A.

[67] Pan Y., Bao H., Sahoo N.G., Wu T., Li L. (2011). Water-soluble poly(*N*-isopropylacrylamide)–graphene sheets synthesized via click chemistry for drug delivery. Adv. Funct. Mater..

[68] Liu Z., Lu G., Li Y., Li Y., Huang X. (2014). Click synthesis of graphene/poly (*N*-(2-hydroxypropyl) methacrylamide) nanocomposite via “grafting-onto” strategy at ambient temperature. RSC Adv..

[69] Yadav S.K., Yoo H.J., Cho J.W. (2013). Click coupled graphene for fabrication of high-performance polymer nanocomposites. J. Polym. Sci. Part B.

[70] Han N.R., Cho J.W. (2016). Click coupled stitched graphene sheets and their polymer nanocomposites with enhanced photothermal and mechanical properties. Compos. Part A.

[71] Mahapatra S.S., Yadav S.K., Cho J.W. (2017). Synthesis of click-coupled graphene sheets with hyperbranched polyurethane: Effective exfoliation and enhancement of nanocomposite properties. J. Appl. Polym. Sci..

[72] Nia A.S., Rana S., Döhler D., Osim W., Binder W.H. (2015). Nanocomposites via a direct graphene-promoted “click”-reaction. Polymer.

[73] Yang X., Zhang X., Ma Y., Huang Y., Wang Y., Chen Y. (2009). Superparamagnetic graphene oxide–Fe_3_O_4_ nanoparticles hybrid for controlled targeted drug carriers. J. Mater. Chem..

[74] Xu Y., Wu Q., Sun Y., Bai H., Shi G. (2010). Three-dimensional self-assembly of graphene oxide and DNA into multifunctional hydrogels. ACS Nano.

[75] Qin X., Guo Z., Liu Z., Zhang W., Wan M., Yang B. (2013). Folic acid-conjugated graphene oxide for cancer targeted chemo-photothermal therapy. J. Photochem. Photobiol. B.

[76] Kiew S.F., Kiew L.V., Lee H.B., Imae T., Chung L.Y. (2016). Assessing biocompatibility of graphene oxide-based nanocarriers: A review. J. Control. Release.

[77] Rubio N., Mei K.-C., Klippstein R., Costa P.M., Hodgins N., Wang J.T.-W., Festy F., Abbate V., Hider R.C., Chan K.L.A. (2015). Solvent-free click-mechanochemistry for the preparation of cancer cell targeting graphene oxide. ACS Appl. Mater. Interfaces.

[78] An Y.-M., Liu T., Tian R., Liu S.-X., Han Y.-N., Wang Q.-Q., Sheng W.-J. (2015). Synthesis of novel temperature responsive PEG-*b*-[PCL-*g*-P (MEO 2 MA-*co*-OEGMA)]-*b*-PEG (tBG) triblock-graft copolymers and preparation of tBG/graphene oxide composite hydrogels via click chemistry. React. Funct. Polym..

[79] Ryu H.J., Mahapatra S.S., Yadav S.K., Cho J.W. (2013). Synthesis of click-coupled graphene sheet with chitosan: Effective exfoliation and enhanced properties of their nanocomposites. Eur. Polym. J..

[80] Wakabayashi K., Pierre C., Dikin D.A., Ruoff R.S., Ramanathan T., Brinson L.C., Torkelson J.M. (2008). Polymer- graphite nanocomposites: Effective dispersion and major property enhancement via solid-state shear pulverization. Macromolecules.

[81] Mazumdar P., Chockalingam S., Rattan S. (2016). Strategy to synthesise nano-engineered polymer nanocomposite with a mechanically strong interface: A highly flexible ammonia gas sensor. RSC Adv..

[82] Dintcheva N.T., Arrigo R., Teresi R., Gambarotti C. (2017). Silanol-POSS as dispersing agents for carbon nanotubes in polyamide. Polym. Eng. Sci..

[83] Bensghaïer A., Salmi Z., Le Droumaguet B., Mekki A., Mohamed A.A., Beji M., Chehimi M.M. (2016). Diazonium interface chemistry and click polymerization: A novel route for carbon nanotube-polytriazole nanocomposites. Surf. Interface Anal..

[84] Inglis A.J., Barner-Kowollik C. (2010). Ultra rapid approaches to mild macromolecular conjugation. Macromol. Rapid Commun..

[85] Nicolaou K.C., Snyder S.A., Montagnon T., Vassilikogiannakis G. (2002). The diels–alder reaction in total synthesis. Angew. Chem. Int. Ed..

[86] Sanyal A. (2010). Diels–alder cycloaddition-cycloreversion: A powerful combo in materials design. Macromol. Chem. Phys..

[87] Tasdelen M.A. (2011). Diels–alder “click” reactions: Recent applications in polymer and material science. Polym. Chem..

[88] Hu X., Meng J. (2005). Effect of organoclay on the curing reactions in bismaleimide/diallyl bisphenol a resin. J. Polym. Sci. Part A.

[89] Hayden H., Gun’ko Y.K., Perova T.S. (2007). Chemical modification of multi-walled carbon nanotubes using a tetrazine derivative. Chem. Phys. Lett..

[90] Sakellariou G., Ji H., Mays J.W., Hadjichristidis N., Baskaran D. (2007). Controlled covalent functionalization of multiwalled carbon nanotubes using [4 + 2] cycloaddition of benzocyclobutenes. Chem. Mater..

[91] Sakellariou G., Ji H., Mays J.W., Baskaran D. (2008). Enhanced polymer grafting from multiwalled carbon nanotubes through living anionic surface-initiated polymerization. Chem. Mater..

[92] Priftis D., Sakellariou G., Hadjichristidis N., Penott E.K., Lorenzo A.T., Müller A.J. (2009). Surface modification of multiwalled carbon nanotubes with biocompatible polymers via ring opening and living anionic surface initiated polymerization. Kinetics and crystallization behavior. J. Polym. Sci. Part A.

[93] Priftis D., Petzetakis N., Sakellariou G., Pitsikalis M., Baskaran D., Mays J.W., Hadjichristidis N. (2009). Surface-initiated titanium-mediated coordination polymerization from catalyst-functionalized single and multiwalled carbon nanotubes. Macromolecules.

[94] Pérez R.A., López J.V., Hoskins J.N., Zhang B., Grayson S.M., Casas M.T., Puiggalí J., Müller A.J. (2014). Nucleation and antinucleation effects of functionalized carbon nanotubes on cyclic and linear poly(ε-caprolactones). Macromolecules.

[95] Chang C.-M., Liu Y.-L. (2009). Functionalization of multi-walled carbon nanotubes with furan and maleimide compounds through diels–alder cycloaddition. Carbon.

[96] Li H.-Y., Chang C.-M., Hsu K.-Y., Liu Y.-L. (2012). Poly(lactide)-functionalized and Fe_3_O_4_ nanoparticle-decorated multiwalled carbon nanotubes for preparation of electrically-conductive and magnetic poly (lactide) films and electrospun nanofibers. J. Mater. Chem..

[97] Wang Y.-H., Chang C.-M., Liu Y.-L. (2012). Benzoxazine-functionalized multi-walled carbon nanotubes for preparation of electrically-conductive polybenzoxazines. Polymer.

[98] Willocq B., Bose R., Khelifa F., Garcia S., Dubois P., Raquez J.-M. (2016). Healing by the joule effect of electrically conductive poly(ester-urethane)/carbon nanotube nanocomposites. J. Mater. Chem. A.

[99] García-García J.M., Bernal M., Verdejo R., López-Manchado M.A., Doncel-Pérez E., Garrido L., Quijada-Garrido I. (2014). Semiconductive bionanocomposites of poly(3-hydroxybutyrate-*co*-3-hydroxyhexanoate) and mwcnts for neural growth applications. J. Polym. Sci. Part B.

[100] Chen Z., Dai X.J., Lamb P.R., de Celis Leal D.R., Fox B.L., Chen Y., du Plessis J., Field M., Wang X. (2012). Practical amine functionalization of multi-walled carbon nanotubes for effective interfacial bonding. Plasma Process. Polym..

[101] Hu L., Hecht D.S., Gruner G. (2010). Carbon nanotube thin films: Fabrication, properties, and applications. Chem. Rev..

[102] Kaur A., Singh I., Kumar J., Bhatnagar C., Dixit S.K., Bhatnagar P.K., Mathur P.C., Covas J.A., da Conceicao Paiva M. (2015). Enhancement in the performance of multi-walled carbon nanotube: Poly(methylmethacrylate) composite thin film ethanol sensors through appropriate nanotube functionalization. Mater. Sci. Semiconduct. Process..

[103] Kuang X., Liu G., Dong X., Wang D. (2016). Enhancement of mechanical and self-healing performance in multiwall carbon nanotube/rubber composites via diels–alder bonding. Macromol. Mater. Eng..

[104] Zhu J., Waengler C., Lennox R.B., Schirrmacher R. (2012). Preparation of water-soluble maleimide-functionalized 3 nm gold nanoparticles: A new bioconjugation template. Langmuir.

[105] Garcia-Astrain C., Ahmed I., Kendziora D., Guaresti O., Eceiza A., Fruk L., Corcuera M., Gabilondo N. (2015). Effect of maleimide-functionalized gold nanoparticles on hybrid biohydrogels properties. RSC Adv..

[106] García-Astrain C., Hernández R., Guaresti O., Fruk L., Mijangos C., Eceiza A., Gabilondo N. (2016). Click crosslinked chitosan/gold nanocomposite hydrogels. Macromol. Mater. Eng..

[107] Liu X., Zhu M., Chen S., Yuan M., Guo Y., Song Y., Liu H., Li Y. (2008). Organic–inorganic nanohybrids via directly grafting gold nanoparticles onto conjugated copolymers through the diels–alder reaction. Langmuir.

[108] García-Astrain C., Chen C., Burón M., Palomares T., Eceiza A., Fruk L., Corcuera M.Á., Gabilondo N. (2015). Biocompatible hydrogel nanocomposite with covalently embedded silver nanoparticles. Biomacromolecules.

[109] Wu C.-S., Kao T.-H., Li H.-Y., Liu Y.-L. (2012). Preparation of polybenzoxazine-functionalized Fe_3_O_4_ nanoparticles through In Situ diels–alder polymerization for high performance magnetic polybenzoxazine/Fe_3_O_4_ nanocomposites. Compos. Sci. Technol..

[110] Engel T., Kickelbick G. (2013). Thermoreversible reactions on inorganic nanoparticle surfaces: Diels–alder reactions on sterically crowded surfaces. Chem. Mater..

[111] Engel T., Kickelbick G. (2014). Self-healing nanocomposites from silica–polymer core–shell nanoparticles. Polym. Int..

[112] Schäfer S., Kickelbick G. (2015). Self-healing polymer nanocomposites based on diels-alder-reactions with silica nanoparticles: The role of the polymer matrix. Polymer.

[113] Chuo T.-W., Liu Y.-L. (2015). Preparation of self-healing organic–inorganic nanocomposites with the reactions between methacrylated polyhedral oligomeric silsesquioxanes and furfurylamine. Compos. Sci. Technol..

[114] Xu Z., Zhao Y., Wang X., Lin T. (2013). A thermally healable polyhedral oligomeric silsesquioxane (POSS) nanocomposite based on diels–alder chemistry. Chem. Commun..

[115] García-Astrain C., Miljevic M., Ahmed I., Martin L., Eceiza A., Fruk L., Corcuera M., Gabilondo N. (2016). Designing hydrogel nanocomposites using TiO_2_ as clickable cross-linkers. J. Mater. Sci..

[116] Arslan M., Gevrek T.N., Lyskawa J., Szunerits S., Boukherroub R., Sanyal R., Woisel P., Sanyal A. (2014). Bioinspired anchorable thiol-reactive polymers: Synthesis and applications toward surface functionalization of magnetic nanoparticles. Macromolecules.

[117] Watson M.A., Lyskawa J.L., Zobrist C.D., Fournier D., Jimenez M., Traisnel M., Gengembre L.O., Woisel P. (2010). A “clickable” titanium surface platform. Langmuir.

[118] Yang Y., Zhu B., Yin D., Wei J., Wang Z., Xiong R., Shi J., Liu Z., Lei Q. (2015). Flexible self-healing nanocomposites for recoverable motion sensor. Nano Energy.

[119] García-Astrain C., González K., Gurrea T., Guaresti O., Algar I., Eceiza A., Gabilondo N. (2016). Maleimide-grafted cellulose nanocrystals as cross-linkers for bionanocomposite hydrogels. Carbohydr. Polym..

[120] Li Y., Louarn G., Aubert P.-H., Alain-Rizzo V., Galmiche L., Audebert P., Miomandre F. (2016). Polypyrrole-modified graphene sheet nanocomposites as new efficient materials for supercapacitors. Carbon.

[121] Li J., Zhang G., Deng L., Zhao S., Gao Y., Jiang K., Sun R., Wong C. (2014). In Situ polymerization of mechanically reinforced, thermally healable graphene oxide/polyurethane composites based on diels–alder chemistry. J. Mater. Chem. A.

[122] Li J., Zhang G., Sun R., Wong C.-P. (2017). A covalently cross-linked reduced functionalized graphene oxide/polyurethane composite based on diels–alder chemistry and its potential application in healable flexible electronics. J. Mater. Chem. C.

[123] Hoyle C.E., Lowe A.B., Bowman C.N. (2010). Thiol-click chemistry: A multifaceted toolbox for small molecule and polymer synthesis. Chem. Soc. Rev..

[124] Lowe A.B. (2014). Thiol-ene “click” reactions and recent applications in polymer and materials synthesis: A first update. Polym. Chem..

[125] Ten Brummelhuis N., Diehl C., Schlaad H. (2008). Thiol–ene modification of 1,2-polybutadiene using UV light or sunlight. Macromolecules.

[126] Rosilo H., Kontturi E., Seitsonen J., Kolehmainen E., Ikkala O. (2013). Transition to reinforced state by percolating domains of intercalated brush-modified cellulose nanocrystals and poly (butadiene) in cross-linked composites based on thiol–ene click chemistry. Biomacromolecules.

[127] Uygun M., Tasdelen M.A., Yagci Y. (2010). Influence of type of initiation on thiol–ene “click” chemistry. Macromol. Chem. Phys..

[128] Parambath Kanoth B., Claudino M., Johansson M., Berglund L.A., Zhou Q. (2015). Biocomposites from natural rubber: Synergistic effects of functionalized cellulose nanocrystals as both reinforcing and cross-linking agents via free-radical thiol–ene chemistry. ACS Appl. Mater. Interfaces.

[129] Fox J.D., Capadona J.R., Marasco P.D., Rowan S.J. (2013). Bioinspired water-enhanced mechanical gradient nanocomposite films that mimic the architecture and properties of the squid beak. J. Am. Chem. Soc..

[130] Schyrr B., Pasche S.P., Voirin G., Weder C., Simon Y.C., Foster E.J. (2014). Biosensors based on porous cellulose nanocrystal–poly(vinyl alcohol) scaffolds. ACS Appl. Mater. Interfaces.

[131] Garber L., Chen C., Kilchrist K.V., Bounds C., Pojman J.A., Hayes D. (2013). Thiol-acrylate nanocomposite foams for critical size bone defect repair: A novel biomaterial. J. Biomed. Mater. Res. Part A.

[132] Riggs B.C., Elupula R., Rehm C., Adireddy S., Grayson S.M., Chrisey D.B. (2015). Click-in ferroelectric nanoparticles for dielectric energy storage. ACS Appl. Mater. Interfaces.

[133] Yang K., Huang X., Zhu M., Xie L., Tanaka T., Jiang P. (2014). Combining raft polymerization and thiol–ene click reaction for core–shell structured polymer@ batio3 nanodielectrics with high dielectric constant, low dielectric loss, and high energy storage capability. ACS Appl. Mater. Interfaces.

[134] Yagci Y., Jockusch S., Turro N.J. (2010). Photoinitiated polymerization: Advances, challenges, and opportunities. Macromolecules.

[135] Hoyle C.E., Lee T.Y., Roper T. (2004). Thiol–enes: Chemistry of the past with promise for the future. J. Polym. Sci. Part A.

[136] Cramer N.B., Scott J.P., Bowman C.N. (2002). Photopolymerizations of thiol–ene polymers without photoinitiators. Macromolecules.

[137] Han J., Zheng Y., Zhao B., Li S., Zhang Y., Gao C. (2014). Sequentially hetero-functional, topological polymers by step-growth thiol-yne approach. Sci. Rep..

[138] Bae J. (2012). Thiol-ene/clay nanocomposite thin film as novel transparent barrier. Polym. Int..

[139] Owusu-Adom K., Schall J., Guymon C.A. (2009). Photopolymerization behavior of thiol–acrylate monomers in clay nanocomposites. Macromolecules.

[140] Kim S.K., Guymon C.A. (2011). Photopolymerization behavior in nanocomposites formed with thiol–acrylate and polymerizable organoclays. J. Polym. Sci. Part A.

[141] Boyd D.A., Naciri J., Fontana J., Pacardo D.B., Shields A.R., Verbarg J., Spillmann C.M., Ligler F.S. (2014). Facile fabrication of color tunable film and fiber nanocomposites via thiol click chemistry. Macromolecules.

[142] Phillips J.P., Mackey N.M., Confait B.S., Heaps D.T., Deng X., Todd M.L., Stevenson S., Zhou H., Hoyle C.E. (2008). Dispersion of gold nanoparticles in UV-cured, thiol–ene films by precomplexation of gold–thiol. Chem. Mater..

[143] Boyd D.A., Bezares F.J., Pacardo D.B., Ukaegbu M., Hosten C., Ligler F.S. (2014). Small-molecule detection in thiol–yne nanocomposites via surface-enhanced raman spectroscopy. Anal. Chem..

[144] Ren F., Yesildag C., Zhang Z., Lensen M.C. (2017). Functional PEG-hydrogels convey gold nanoparticles from silicon and aid cell adhesion onto the nanocomposites. Chem. Mater..

[145] Mardare L., Benea L. (2017). Development of Anticorrosive Polymer Nanocomposite Coating for Corrosion Protection in Marine Environment.

[146] Nazari M.H., Shi X. (2016). Polymer-based nanocomposite coatings for anticorrosion applications. Industrial Applications for Intelligent Polymers and Coatings.

[147] Majumdar D., Blanton T.N., Schwark D.W. (2003). Clay–polymer nanocomposite coatings for imaging application. Appl. Clay Sci..

[148] Bae J., Lee J., Park C.S., Kwon O.S., Lee C.-S. (2016). Fabrication of photo-crosslinkable polymer/silica sol–gel hybrid thin films as versatile barrier films. J. Ind. Eng. Chem..

[149] Mülazim Y., Çakmakçi E., Kahraman M.V. (2013). Properties of thiol–ene photocurable highly hydrophobic and oleophobic nanocomposite coatings on abs and hips substrates. Adv. Polym. Technol..

[150] Chollet B., Li M., Martwong E., Bresson B., Fretigny C., Tabeling P., Tran Y. (2016). Multiscale surface-attached hydrogel thin films with tailored architecture. ACS Appl. Mater. Interfaces.

[151] Li S., Qiu S., Yu B., Tang G., Xing W., Hu Y. (2016). Poss-functionalized polyphosphazene nanotube: Preparation and effective reinforcement on uv-curable epoxy acrylate nanocomposite coatings. RSC Adv..

[152] Wang X., Wang X., Song L., Xing W., Tang G., Hu W., Hu Y. (2013). Preparation and thermal stability of UV-cured epoxy-based coatings modified with octamercaptopropyl poss. Thermochim. Acta.

[153] Acar S.B., Ozcelik M., Uyar T., Tasdelen M.A. (2017). Polyhedral oligomeric silsesquioxane-based hybrid networks obtained via thiol-epoxy click chemistry. Iran. Polym. J..

[154] Zhao F., Huang Y. (2011). Preparation and properties of polyhedral oligomeric silsesquioxane and carbon nanotube grafted carbon fiber hierarchical reinforcing structure. J. Mater. Chem..

[155] Zhao F., Huang Y., Liu L., Bai Y., Xu L. (2011). Formation of a carbon fiber/polyhedral oligomeric silsesquioxane/carbon nanotube hybrid reinforcement and its effect on the interfacial properties of carbon fiber/epoxy composites. Carbon.

[156] Tzur-Balter A., Shatsberg Z., Beckerman M., Segal E., Artzi N. (2015). Mechanism of erosion of nanostructured porous silicon drug carriers in neoplastic tissues. Nat. Commun..

[157] Ferreira M.P., Ranjan S., Correia A.M., Mäkilä E.M., Kinnunen S.M., Zhang H., Shahbazi M.-A., Almeida P.V., Salonen J.J., Ruskoaho H.J. (2016). In Vitro and In Vivo assessment of heart-homing porous silicon nanoparticles. Biomaterials.

[158] Poole L.B. (2015). The basics of thiols and cysteines in redox biology and chemistry. Free Radic. Biol. Med..

[159] Shahbazi M.-A., Almeida P.V., Correia A., Herranz-Blanco B., Shrestha N., Mäkilä E., Salonen J., Hirvonen J., Santos H.A. (2017). Intracellular responsive dual delivery by endosomolytic polyplexes carrying DNA anchored porous silicon nanoparticles. J. Control. Release.

[160] Feld A., Koll R., Fruhner L.S., Krutyeva M., Pyckhout-Hintzen W., Weiß C., Heller H., Weimer A., Schmidtke C., Appavou M.-S. (2017). Nanocomposites of highly monodisperse encapsulated superparamagnetic iron oxide nanocrystals homogeneously dispersed in a poly(ethylene oxide) melt. ACS Nano.

[161] Asmussen S.V., Vallo C.I. (2016). Facile preparation of silver-based nanocomposites via thiol-methacrylate ‘click’photopolymerization. Eur. Polym. J..

[162] Jin F., Zheng M.-L., Zhang M.-L., Zhao Z.-S., Duan X.-M. (2014). A facile layer-by-layer assembly method for the fabrication of fluorescent polymer/quantum dot nanocomposite thin films. RSC Adv..

[163] Salavagione H.J. (2014). Promising alternative routes for graphene production and functionalization. J. Mater. Chem. A.

[164] Luong N.D., Sinh L.H., Johansson L.S., Campell J., Seppälä J. (2015). Functional graphene by thiol-ene click chemistry. Chem. Eur. J..

[165] Castelaín M., Martínez G., Marco C., Ellis G., Salavagione H.J. (2013). Effect of click-chemistry approaches for graphene modification on the electrical, thermal, and mechanical properties of polyethylene/graphene nanocomposites. Macromolecules.

[166] Yu B., Wang X., Xing W., Yang H., Wang X., Song L., Hu Y., Lo S. (2013). Enhanced thermal and mechanical properties of functionalized graphene/thiol-ene systems by photopolymerization technology. Chem. Eng. J..

[167] Yu B., Shi Y., Yuan B., Liu L., Yang H., Tai Q., Lo S., Song L., Hu Y. (2015). Click-chemistry approach for graphene modification: Effective reinforcement of UV-curable functionalized graphene/polyurethane acrylate nanocomposites. RSC Adv..

[168] Choi J.-Y., Kim S.W., Cho K.Y. (2014). Improved thermal conductivity of graphene encapsulated poly(methyl methacrylate) nanocomposite adhesives with low loading amount of graphene. Compos. Sci. Technol..

[169] Liras M., García O., Quijada-Garrido I., Ellis G., Salavagione H.J. (2014). Homogenous thin layer coated graphene via one pot reaction with multidentate thiolated pmmas. J. Mater. Chem. C.

[170] Castelaín M., Martínez G., Ellis G., Salavagione H.J. (2013). A versatile chemical tool for the preparation of conductive graphene-based polymer nanocomposites. Chem. Commun..

[171] Zhang Y., Li Q., Wang W., Guo A., Li J., Li H. (2017). Efficient and robust reactions for polyethylene covalently grafted carbon nanotubes. Macromol. Chem. Phys..

[172] Su Z., Liu Y., Xie Q., Chen L., Zhang Y., Meng Y., Li Y., Fu Y., Ma M., Yao S. (2012). Preparation of thiolated polymeric nanocomposite for sensitive electroanalysis of dopamine. Biosens. Bioelectron..

[173] Liu Y., Su Z., Zhang Y., Chen L., Gu T., Huang S., Liu Y., Sun L., Xie Q., Yao S. (2013). Amperometric determination of ascorbic acid using multiwalled carbon nanotube-thiolated polyaniline composite modified glassy carbon electrode. J. Electroanal. Chem..

[174] Su Z., Liu Y., Zhang Y., Xie Q., Chen L., Huang Y., Fu Y., Meng Y., Li X., Ma M. (2013). Thiol–ene chemistry guided preparation of thiolated polymeric nanocomposite for anodic stripping voltammetric analysis of Cd^2+^ and Pb^2+^. Analyst.

[175] Lee J.E., Lee D.J., Lee N., Kim B.H., Choi S.H., Hyeon T. (2011). Multifunctional mesoporous silica nanocomposite nanoparticles for ph controlled drug release and dual modal imaging. J. Mater. Chem..

[176] De France K.J., Chan K.J., Cranston E.D., Hoare T. (2016). Enhanced mechanical properties in cellulose nanocrystal-poly(oligoethylene glycol methacrylate) injectable nanocomposite hydrogels through control of physical and chemical cross-linking. Biomacromolecules.

[177] Liu D., Zhang H., Mäkilä E., Fan J., Herranz-Blanco B., Wang C.-F., Rosa R., Ribeiro A.J., Salonen J., Hirvonen J. (2015). Microfluidic assisted one-step fabrication of porous silicon@ acetalated dextran nanocomposites for precisely controlled combination chemotherapy. Biomaterials.

[178] Knall A.-C., Slugovc C. (2013). Inverse electron demand diels–alder (iedda)-initiated conjugation: A (high) potential click chemistry scheme. Chem. Soc. Rev..

[179] Yuan Y., Liang G. (2014). A biocompatible, highly efficient click reaction and its applications. Org. Biomol. Chem..

